# A Modified Single Metamaterial Split-Ring Resonator for Enhanced Sensitivity

**DOI:** 10.3390/s26144659

**Published:** 2026-07-22

**Authors:** Amal Swileh, Rola Saad, Salam K. Khamas

**Affiliations:** 1 School of Electrical and Electronic Engineering, University of Sheffield, Sheffield S10 2TN, UK; s.khamas@sheffield.ac.uk; 2 Medical Physics and Biomedical Engineering, University College London, London WC1E 6BT, UK; r.saad@ucl.ac.uk

**Keywords:** asymmetric split resonator, CST, glucose level, metamaterial, microwave sensing, rectangular waveguide

## Abstract

A novel microwave biosensor operating in the C-band is developed and characterised for enhanced glucose sensing applications. The sensor is based on a single metamaterial asymmetric split-ring resonator (SASR) and has been investigated in two configurations: a single semi-circular design (SASR-S) and a double semi-circular design (SASR-D). The structural modifications were introduced to enlarge the sensing area by creating two high-field hotspots, thereby increasing the interaction between the electromagnetic (EM) field and the sample, which consequently enhances the overall sensor sensitivity. The sensor is fabricated on a Rogers AD350A substrate and is optimised to detect glucose levels in a 1 µL solution applied within each semi-circle sensing region. To characterise the sensor’s enhanced sensitivity, we performed a 3D electromagnetic simulation of a small droplet positioned within a semicircular sensing region, varying the relative permittivity of the droplet from 45 to 65. The resulting shifts in resonant frequency served as a primary indicator of dielectric sensitivity. The sensor’s response was experimentally validated using a vector network analyser to measure the transmission coefficient (S_21_) of samples with no glucose and at glucose concentrations of 97 mg/dL to 286 mg/dL. The results demonstrate that the resonator configuration strongly influences the resonance frequency shift and sensitivity, with the SASR-D configuration being the most effective design. This has also been confirmed by measurements demonstrating a sensitivity of approximately 2.4 MHz/(mg/dL), representing an approximately two-fold improvement over the SASR-S sensor (sensitivity: 1.27 MHz/(mg/dL)) and a notable enhancement over previously reported sensors. These findings demonstrate the practical potential of the proposed sensor for blood glucose monitoring applications.

## 1. Introduction

Diabetes is a widespread health issue that affects millions of people around the world. It is a long-term condition that disrupts the body’s ability to manage blood sugar, often leading to persistently high glucose levels. Effective disease management requires regular monitoring of blood sugar, which is vital for individuals living with diabetes. This condition poses a significant public health challenge due to its links to reduced life expectancy and considerable financial and emotional strain on patients, families, and healthcare systems [1]. Diabetes develops either when the pancreas fails to produce adequate insulin or when the body’s cells are unable to utilise insulin effectively, with both mechanisms resulting in hyperglycaemia. Persistently high blood glucose levels can lead to serious complications, affecting various organs and systems, including the lower limbs, cardiovascular system, eyes, and kidneys, and may result in life-threatening outcomes such as myocardial infarction and severe infections.

There are two primary forms of diabetes: Type 1 diabetes, which is caused by inadequate insulin production, and Type 2 diabetes, which arises from impaired insulin utilisation by the body’s cells. Type 2 diabetes is the most widespread form worldwide, and it accounts for over 85% of all cases, highlighting its prevalence compared to Type 1 [1]. When blood glucose levels drop below 70 mg/dL, the condition is known as hypoglycaemia. In contrast, hyperglycaemia occurs when blood glucose levels rise above 120 mg/dL during fasting or exceed 180 mg/dL after meals [2]. Continuous glucose monitoring devices are crucial for preventing diabetes-related complications. There are various devices available, including invasive, minimally invasive and non-invasive devices. Invasive techniques use self-monitoring blood glucose devices that require finger pricking. Minimally invasive devices [3] involve implanting a thin sensor beneath the skin to measure interstitial fluid. Non-invasive devices [4] operate without the need for a fingerstick or fluid sample. Several methods have been studied for monitoring glucose levels, including electrical [4], optical [5], thermal [6] and radio frequency techniques [7].

Cebedio et al. [8] suggested a glucose-monitoring antenna sensor based on a coplanar waveguide with a ground plane (GCPW), operating at 1.8 GHz, using an FR4 substrate (1.6 mm thickness, 4.3 dielectric constant). The sensor exhibited a frequency shift of 31.2 MHz for dielectric constants ranging from 30 to 70, with a corresponding variation in the quality factor by 202 units. Esraa et al. [9] designed a single-slot defected ground structure (DGS) on Rogers RO4003C substrate (with 0.813 mm thickness & 3.38 dielectric constant) to measure blood concentration at 2.4 GHz. In [10], an open-loop sensor was designed to leverage the strong electric field intensity between the resonator’s open ends, enhancing sensitivity by positioning the sample at the open-end gap. Another study [11] utilised three resonant cells with single split rings operating between 3–4 GHz to monitor glucose levels from 100 to 300 mg/dL, achieving higher sensitivity through the strong coupling between adjacent cells. Moreover, another study [12] investigated a millimetre-wave sensor for glucose detection, incorporating two small slabs as absorbers to mitigate signal interference and improve electric field distribution, effectively doubling the sensor’s sensitivity.

High-quality factor resonances in metamaterial resonators enhance the interaction between EM waves and the material under test (MUT). These interactions reveal critical information about the material’s properties, such as its dielectric characteristics or glucose concentration, through frequency shifts. Therefore, researchers have proposed various compact metamaterial structures due to their ability to achieve a high-quality factor, which improves sensor performance and sensitivity [13,14,15]. Omer et al. [13] achieved high sensitivity using a honeycomb-shaped complementary split ring resonator (CSSR). Further, a double complementary split ring resonator (DCSSR) was developed in [16]. Even stronger sensitivity and a high-quality factor were introduced using a metamaterial asymmetric single split resonator (ASSR) [17]. Asymmetric unit structures are incorporated into resonators to achieve sharp resonances with high quality factors by minimising radiation losses [18].

The Single Asymmetric Split Resonator (SASR) sensor, as detailed in [19], functions by leveraging electromagnetic wave interactions with the surrounding medium. A key feature of the sensor’s structure is an asymmetric resonator that exhibits Fano resonance, a phenomenon characterised by the interference between a distinct narrowband state and a broad continuum, resulting in a pronounced spectral response and enhanced field confinement. The high-quality factor associated with this resonance is attributed to a substantial reduction in the effective speed of light, driven by rapid fluctuations in phase velocity across frequencies [19].

In this paper, a microwave resonator biosensor operating within the C-band has been developed utilising two geometric configurations to evaluate its resonator characteristics and sensitivity for glucose detection. Specifically, two design variants were analysed: a baseline SASR-S sensor [19], and an enhanced configuration featuring double semi-circular elements (SASR-D).

The proposed sensor is intended for invasive glucose measurements, where direct interaction between the sensing element and the biological sample provides reliable and accurate measurements. Although considerable progress has been made in non-invasive glucose monitoring, these systems often require periodic calibration to maintain accuracy. Consequently, invasive microwave sensors can serve as reliable reference devices, providing high-precision measurements for applications that require direct sample analysis.

Despite the excellent sensitivity achieved by ASSR-based microwave sensors, previously reported designs have typically utilised only a single sensing hotspot, while the second inherent gap of the asymmetric resonator remained unused [19]. To address this limitation, this work introduces a dual semi-circular asymmetric split-ring resonator (SASR-D), in which both gap regions are functionalised as active sensing sites. Unlike previously reported approaches that improve sensitivity by increasing the sensing area through multi-cell arrays or duplicated resonators [11,20,21]. The proposed strategy preserves the original resonator footprint while simultaneously exploiting two high-electric-field interaction regions within the same compact structure. As a result, the sensor maintains a small physical size, requires only 2 μL of sample volume (1 μL per sensing region), and retains the simple fabrication process of the original SASR geometry.

The aim of this work is to develop and experimentally validate a compact microwave biosensor that enhances glucose-sensing sensitivity without increasing the resonator footprint or structural complexity. To achieve this objective, a novel dual semi-circular asymmetric split-ring resonator (SASR-D) is proposed, in which both inherent gap regions are utilised as active sensing hotspots. The performance of the proposed sensor is evaluated through numerical simulations and compared with that of the conventional single semi-circular configuration (SASR-S). Experimental measurements at three representative glucose concentrations (97, 182, and 286 mg/dL) are conducted as a proof-of-concept validation to verify the enhanced sensitivity of the proposed SASR-D configuration.

## 2. Background Theory: Dielectric Properties of Glucose Solutions

The frequency-dependent complex permittivity of glucose–water solutions can be described by the first-order Debye relaxation model, originally proposed by Hofmann et al. [22], who measured several glucose–water solutions of varying concentrations using a commercial open-ended coaxial probe over a broadband frequency range. The model takes the form:

(1) $$\hat{\varepsilon }\left(\omega ,x\right)=\;{\overset{\prime }{\varepsilon }}_{c}\left(x\right)-\;j{\overset{\prime \prime }{\varepsilon }}_{c}\left(x\right)=\varepsilon_{\infty }(x)+\;\frac{\varepsilon_{stat}\left(x\right)-\;\varepsilon_{\infty }\left(x\right)}{1+\;j\omega \tau \left(x\right)}\;\omega =2\pi f$$

where:

ω: Angular frequency;

${\overset{\prime }{\varepsilon }}_{c}\left(x\right)$: The dielectric constant;

${\overset{\prime \prime }{\varepsilon }}_{c}(x)$: The dielectric loss;

$\varepsilon_{\infty }$: The permittivity at high frequencies;

$\varepsilon_{stat}$: The static permittivity at low frequencies;

τ: The relaxation time constant of the medium;

x: The glucose concentration.

From these, the real part of the complex permittivity, the dielectric constant, is derived as follows:

Real part:

(2) $$\varepsilon^{\prime }=\;\varepsilon_{\infty }+\;\frac{\varepsilon_{stat}-\;\varepsilon_{\infty }}{1+\;{\left(\omega \tau \right)}^{2}}$$

and the imaginary part, representing dielectric loss, is

(3) $$\varepsilon^{\prime \prime }=\frac{{(\varepsilon }_{stat}-\varepsilon_{\infty })\;\omega \tau }{1+{\left(\omega \tau \right)}^{2}}$$

(4) $$Loss\;tangent\;(tan\;\delta )=\frac{\varepsilon^{\prime \prime }}{\varepsilon^{\prime }}$$

The resonance frequency shift is primarily governed by changes in real part $\varepsilon^{\prime }$, whilst the imaginary part $\varepsilon^{\prime \prime }$ principally influences the resonance depth and Q-factor rather than the frequency position.

## 3. SASR Sensor Design and Operating Principle

The proposed sensor is based on SASR operating at approximately 7.6 GHz. The SASR geometry produces Fano resonance, resulting in a sharp, asymmetric spectral response with a high Q-factor, defined as

(5) $$Q=\frac{f_{0}}{∆f_{3dB}}$$

The sensing mechanism relies on placing the glucose sample in the high-electric-field region, typically concentrated at the split gap, where the field is strongly confined. Variations in glucose concentration alter the permittivity, leading to a corresponding change in the resonator’s effective capacitance, which in turn causes a measurable shift in the resonance frequency. Consequently, glucose concentration is determined indirectly through the relationship between permittivity variation, capacitance change, and resonance frequency shift. From an equivalent-circuit perspective, the split gaps serve as the dominant capacitive elements of the resonator, while the metallic ring provides the corresponding inductive component. Since the electric field is strongly concentrated within the gap regions, these capacitive elements store most of the electric energy. When the glucose sample is introduced into the semicircular sensing region, its dielectric permittivity modifies the effective gap capacitance, thereby perturbing the LC resonance and producing the observed shift in resonance frequency. Consequently, the magnitude of the frequency shift is directly related to the strength of the electric-field interaction between the sample and the sensing hotspot.

**Figure 1:**
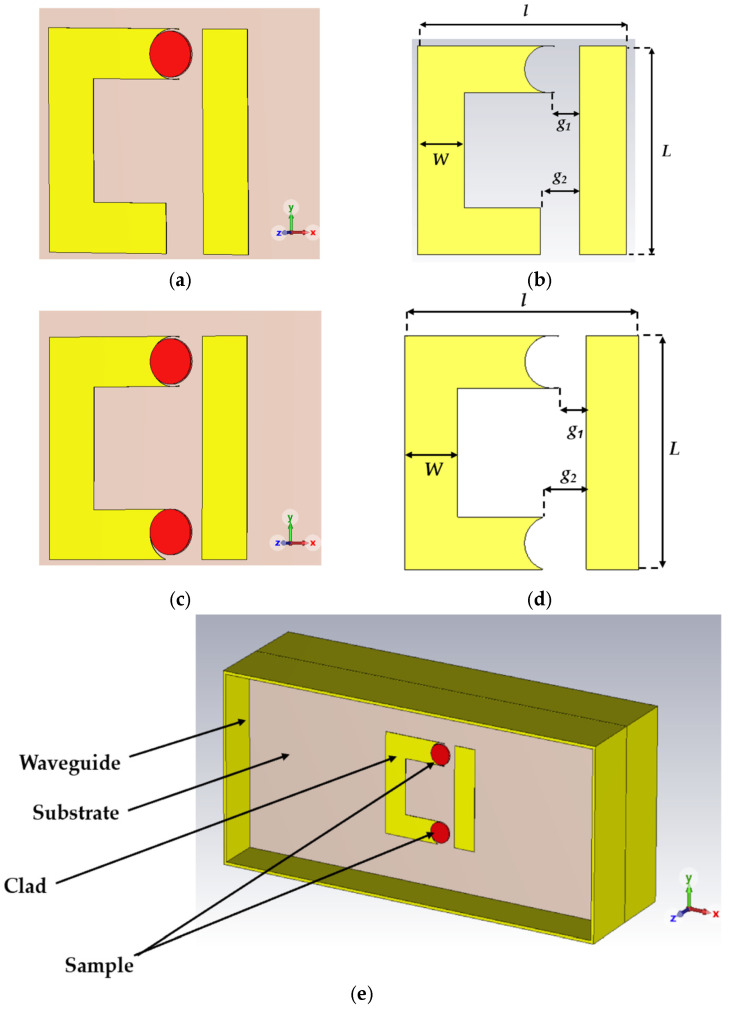
SASR sensor: (**a**) With single semi-circle and droplet, (**b**) With single semi-circle but without droplet, (**c**) With double semi-circle and droplets, (**d**) With double semi-circle but without droplet, (**e**) 3-D SASR sensor with double semi-circle (with droplets). The sensor dimensions are *L* = 8.5 mm, *l* = 8.5 mm, *W* = 1.91 mm, gap *g*_1_ = 1.02 mm, *g*_2_ = 1.6 mm, and the diameter of the semicircle is 1.9 mm.

The SASR-S sensor, with and without the sample, is illustrated in Figure 1a and Figure 1b, respectively, while the SASR-D sensor, with and without the sample, is shown in Figure 1c and Figure 1d, respectively. A 3D representation of the SASR-D structure is presented in Figure 1e.

Comparative analysis of these designs was conducted to assess their sensing capabilities and practical viability for glucose monitoring applications. The resonator structure consists of two gaps; in the first configuration, only one gap end is terminated with a semi-circular structure, whereas in the second configuration, both gap ends are terminated with semi-circular structures. The semi-circular resonator is preferable to a full circular design because it provides stronger electric field concentration within the sensing region, resulting in greater interaction with the sample, whereas in a full circular resonator, the electric field is largely distributed outside the structure [23]. The proposed sensor was designed to operate at 7.6 GHz. According to the results reported in [24], operating at 7 GHz provides higher sensitivity than lower operating frequencies. The choice of a Rogers AD350A substrate is due to its availability and its ability to provide a high Q-factor, which results in a narrow bandwidth and, in turn, leads to higher sensitivity and selectivity [24]. The main advantage of the proposed sensor lies in its simple, cost-effective structure and its ability to detect small sample volumes while maintaining high sensitivity.

The introduction of a second semi-circular element is intended to enhance the sensor sensitivity by increasing the overall resonance frequency shift. In the SASR-S sensor, the sensing mechanism is limited to one active region with a single sample droplet. In contrast, the SASR-D sensor introduces an additional sensing region, allowing two sample droplets (each approximately 1 µL) to interact simultaneously with the high-electric-field regions. This effectively increases the resonator’s dielectric loading, leading to a larger perturbation and, consequently, a more pronounced resonance-frequency shift. As a result, the SASR-D sensor improves the detectability of small changes in permittivity and enhances overall sensitivity.

All designs have dimensions of *L × l = 0.3 λ_eff_ × 0.3 λ_eff_*

where *λ_eff_* is

(6) $$\lambda_{eff}=\frac{\lambda_{0}}{\sqrt{\varepsilon_{eff}}}=\frac{c}{f_{r}\sqrt{\frac{\varepsilon_{r}+1}{2}}}$$

where:

$\lambda_{eff}$: The effective wavelength of the wave inside the resonator.

$\lambda_{0}$: The free-space wavelength.

c: The speed of light in free space.

$f_{r}$: The resonant frequency.

$\varepsilon_{eff}$: The effective permittivity of the medium. It accounts for the fact that the wave is partly in the dielectric and partly in air.

$\varepsilon_{r}:$ The relative permittivity (dielectric constant) of the substrate material.

A Rogers AD350A substrate with a relative permittivity of 3.5, a loss tangent of 0.003, and a thickness of 0.762 mm was used. The geometrical dimensions of the clad for both sensors were *L* = 8.5 mm, *l* = 8.5 mm, *W* = 1.91 mm, gap *g*_1_ = 1.02 mm, *g*_2_ = 1.6 mm, and the diameter of the semicircle was 1.9 mm.

### 3.1. Equivalent Circuit Model

**Figure 2:**
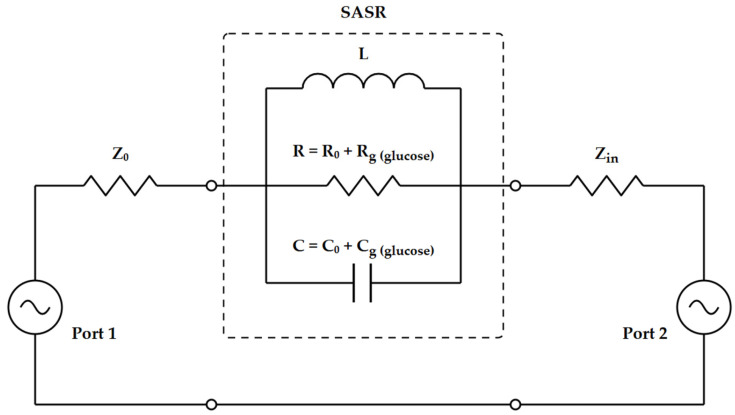
The equivalent circuit of SASR sensors.

In the quasi-static limit, the SASR system within the waveguide can be approximated by three fundamental lumped elements connected in parallel [19,25], as illustrated in Figure 2. The copper trace of the SASR contributes to the inductance *L*, while losses associated with the metallic and substrate materials are represented by the total resistance *R*, which can therefore be expressed as

*R* = *R*_0_ + *R_g_*_(*glucose*)_ = *R*_0_ + *R_g_*_1(*glucose*)_ + *R_g_*_2(*glucose*)_ (7)

The capacitive behaviour of the resonator, primarily governed by the gap regions, can be expressed as

*C* = *C*_0_ + *C_g_*_(*glucose*)_ = *C*_0_ + *C_g_*_1(*glucose*)_ + *C_g_*_2(*glucose*)_ (8)

where R_0_ and C_0_ denote the intrinsic resistance and capacitance in the absence of any glucose solution, respectively. The intrinsic resistance of the resonator primarily arises from ohmic losses in the metallic parts of the structure, particularly the copper trace of the SASR. It also includes dielectric losses, which arise from energy absorption in the substrate and depend on its loss tangent (tan δ). The intrinsic capacitance represents the inherent electrical energy storage between the metallic elements of the resonator (such as the split gaps or semicircular arms) and the dielectric substrate. *R_g_*_1(glucose)_, *R*_g2(glucose)_, *C*_g1(glucose)_, and *C*_g2(glucose)_ represent the resistive and capacitive effects when glucose droplets are placed within the semicircular regions.

Accordingly, from the equivalent circuit shown in Figure 2, the admittance of the parallel RLC circuit is given by

(9) $$Y\left(\omega \right)=\frac{1}{R}+j\omega C+\frac{1}{j\omega L}$$

which can be rewritten by substitution as

(10) $$Y\left(\omega \right)=\frac{1}{R_{0}+R_{g}}+j\omega \left(C_{0}+C_{g}\right)-\frac{j}{\omega L}$$

The corresponding input impedance of the system is obtained as the inverse of the admittance, such that

(11) $$Z(\omega )=\frac{1}{Y\left(\omega \right)}$$

The resonance condition is achieved when the imaginary component of the admittance becomes zero, yielding $\omega \left(C_{0}+C_{g}\right)-\frac{1}{\omega L}\;=\;0$. Solving this expression leads to the resonance frequency:

(12) $$\omega_{0}=\frac{1}{\sqrt{L{(C}_{0}+C_{g})}}$$

or equivalently

(13) $$f_{0}=\frac{1}{2\pi \sqrt{L{(C}_{0}+C_{g})}}$$

This relationship indicates that variations in glucose concentration directly influence the effective capacitance *Cg*, resulting in an increase in the total capacitance of the resonator. Consequently, the resonance frequency decreases as the glucose concentration increases, which forms the fundamental sensing mechanism of the SASR sensor.

### 3.2. Simulation and Experimental Setup

Full-wave electromagnetic simulations were performed using CST Microwave Studio, with waveguide port excitation corresponding to a WR137 rectangular waveguide.

To characterise the sensor response across the clinically relevant range of blood glucose states, simulations were conducted using discrete values of the real permittivity ε′ spanning from 45 to 65. This range was selected based on the known dielectric behaviour of blood at 7.6 GHz. Specifically, the relative permittivity of normal human blood at 7.6 GHz has been reported as approximately 49 [26], corresponding to normoglycemic conditions with a fasting glucose level of 4–6 mmol/L (72–108 mg/dL). As blood glucose rises into the hyperglycaemic range, ε′ decreases below 49, because elevated glucose molecules bind to free water molecules and reduce the number of unbound dipoles available for electromagnetic coupling, thereby lowering the dielectric response of the medium [27,28]. Conversely, in hypoglycaemic conditions, ε′ rises above 49, as the reduced glucose concentration leaves a greater proportion of water molecules unbound and freely rotating, increasing the dipolar contribution to permittivity [27]. It should be noted that the value ε′ ≈ 49 is not a universal constant; it varies with blood composition, haematocrit, temperature, and measurement technique [29].

Therefore, the simulated permittivity range of 45–65 enables a comprehensive evaluation of sensor sensitivity. Both the SASR-S and SASR-D sensors were simulated across this range for direct comparison.

For experimental validation, the fabricated resonators on Rogers AD350A substrate were mounted within a WR137 rectangular waveguide assembly. Mechanical stability was ensured by introducing mounting holes in the substrate and securing it using nylon screws, nuts, and washers. The substrate dimensions were precisely matched to the waveguide cross-section to guarantee accurate alignment and a repeatable measurement position. All measurements were conducted at a controlled room temperature of 25 °C, which is a critical experimental parameter given the well-documented temperature dependence of the dielectric permittivity of aqueous glucose solutions at microwave frequencies [29]. S_21_ measurements were acquired using an Agilent E5071B vector network analyser (VNA), with the waveguide system calibrated using a Thru-Reflect-Line (TRL) kit prior to all measurements. Glucose solutions were prepared by dissolving glucose powder in distilled water; concentrations of 97 mg/dL and 286 mg/dL were used, with values verified using a commercial glucometer. A precision 1 µL syringe was used to apply the solution to each sensing region. Between successive measurements, the resonator surface was thoroughly rinsed with distilled water to remove residual contamination and ensure measurement integrity. The principal measurement apparatus comprised two right-angled rectangular waveguide sections, the fabricated resonator substrate, a calibrated VNA, a commercial glucometer for concentration verification, and a precision micro-syringe for sample application.

## 4. Results and Discussion

This section presents both the simulation results and the experimental proof-of-concept validation of the proposed microwave biosensor. The simulations are used to investigate the sensor response over a broad range of dielectric permittivity, whereas the experimental measurements are performed at three representative glucose concentrations to verify the sensing mechanism and validate the predicted improvement in sensitivity.

**Figure 3:**
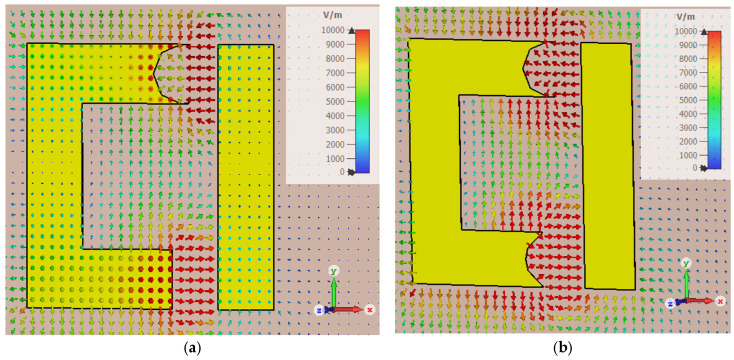
Electric field (E-field) distribution for different resonator configurations: (**a**) sensor with a single semi-circular element, and (**b**) sensor with double semi-circular elements.

The semi-circular geometry used in the sensor design plays a critical role in enhancing electric-field (E-field) confinement within the sensing region. To further amplify the field intensity, a second semi-circular element was introduced in the lower section of the structure. This design modification was evaluated using electromagnetic simulations by comparing the E-field distributions of configurations with one and two semi-circular elements, as shown in Figure 3a and Figure 3b, respectively. The simulation results indicate that the addition of a second semi-circular sensing region significantly enhances the electric-field confinement in this area.

The peak electric-field intensity increased from 25 × 10^3^ V/m in the SASR-S configuration to 31 × 10^3^ V/m in the SASR-D configuration, corresponding to a 1.24-fold increase. The glucose solution is applied directly to the semi-circular region, where strong E-field confinement facilitates pronounced interaction between the electromagnetic field and the sample. This interaction enhances the sensor’s sensitivity to concentration variations.

### 4.1. SASR-D Sensor

**Figure 4:**
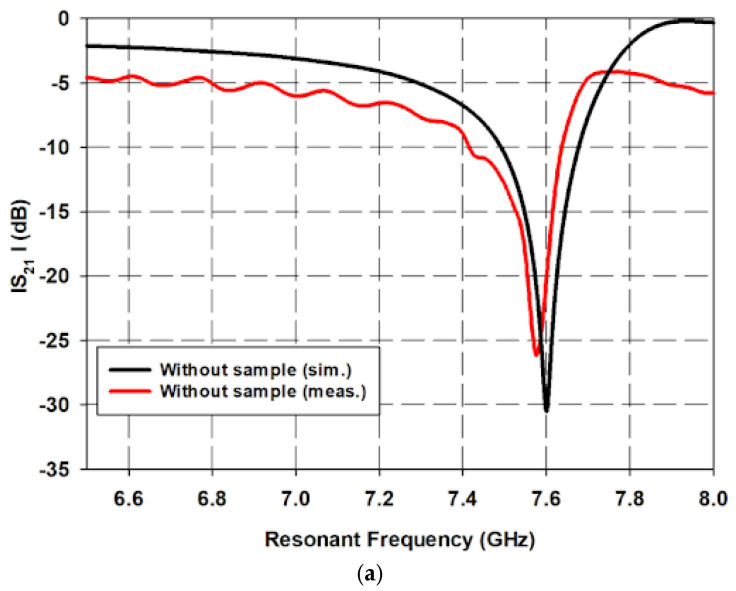

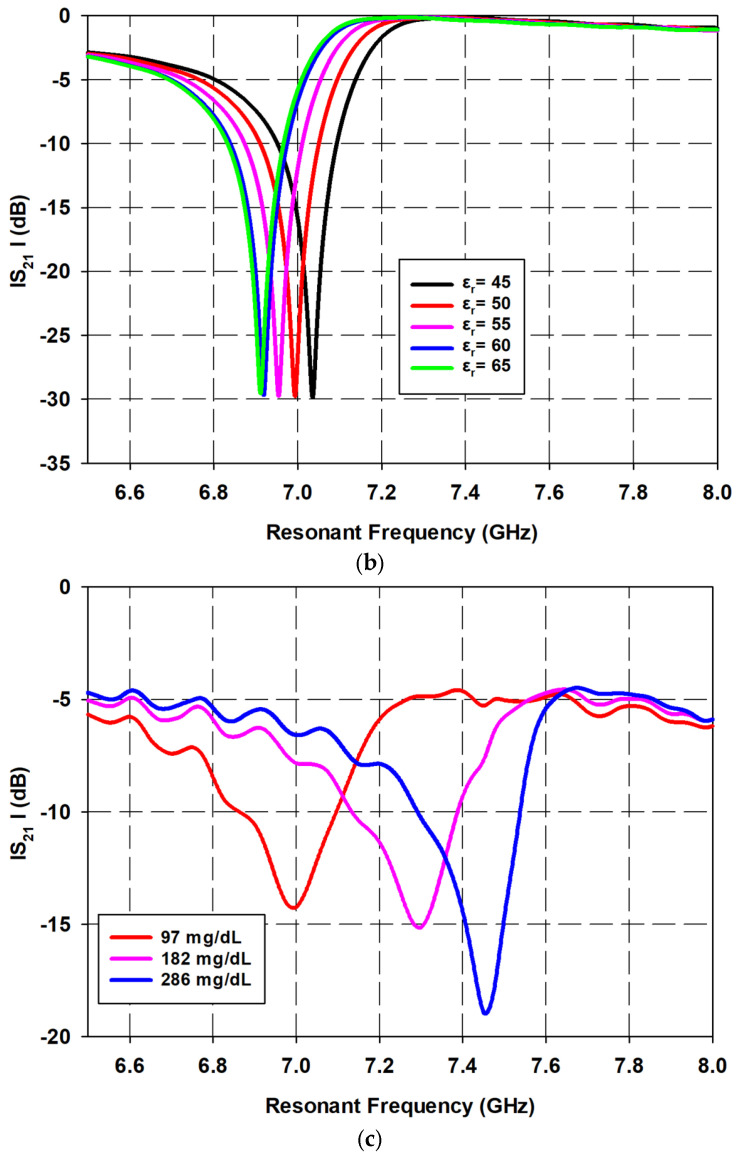
(**a**) The simulated and measured S21 of the sensor (unloaded). (**b**) The simulated S21 of the Sensor with different permittivity. (**c**) The measured S_21_ of the Sensor with different glucose concentration.

The double semi-circular resonator configuration was evaluated using a full-wave electromagnetic simulation. As illustrated in Figure 4a, the unloaded resonator exhibits a sharply defined resonance peak at 7.6 GHz with a narrow bandwidth, yielding a high Q-factor of 408, calculated using Equation (5). This high-Q response indicates minimal energy loss and strong electric-field confinement within the sensing region.

Full-wave electromagnetic simulations were performed to predict the sensor response over a wide range of dielectric permittivity values and to evaluate the expected resonance-frequency shift under controlled conditions. Simulations were conducted across a range of real permittivity values from *ε′* = 45 to *ε′* = 65. The simulation results reveal a resonance frequency shift from 6.91 GHz to 7.04 GHz, corresponding to a total shift of 130 MHz, as demonstrated in Figure 4b.

To provide proof-of-concept experimental validation of the proposed sensor, measurements were conducted using glucose–water solutions at three representative glucose concentrations (97, 182, and 286 mg/dL). These measurements were intended to verify the sensing mechanism and confirm the sensitivity enhancement predicted by the simulations rather than to provide a comprehensive experimental characterisation. Under unloaded conditions, the resonant frequency was measured at 7.58 GHz, corresponding to a 0.26% deviation from the simulated value, as illustrated in Figure 4a. Glucose solutions of varying concentrations were applied to both semicircular regions of the resonator to ensure direct interaction with the sensing area. The addition of a 1 µL droplet perturbed the electric field in the sensitive region of the dielectric substrate, resulting in a further shift in S_21_ due to changes in the effective permittivity of the medium surrounding the resonator.

To avoid the need for human participant involvement and associated ethical approval requirements, all measurements were conducted using prepared glucose–water solutions rather than biological samples obtained from volunteers. As shown in Figure 4c, glucose levels ranging from 97 mg/dL to 286 mg/dL produced a frequency shift from 6.99 GHz to 7.45 GHz, corresponding to a 460 MHz shift. Increasing glucose concentration is associated with a lower dielectric constant, which in turn causes an upward shift in the resonant frequency [9,28,30]. A damping effect was evident in the resonance peaks due to the lossy nature of the glucose–water mixture, reducing the resonance depth. The proposed sensor achieved a sensitivity of 2.4 MHz/(mg/dL). Although only three glucose concentrations were experimentally investigated, the measured resonance-frequency shifts follow the same trend predicted by the electromagnetic simulations, thereby providing proof-of-concept validation of the proposed sensing approach.

**Figure 5:**
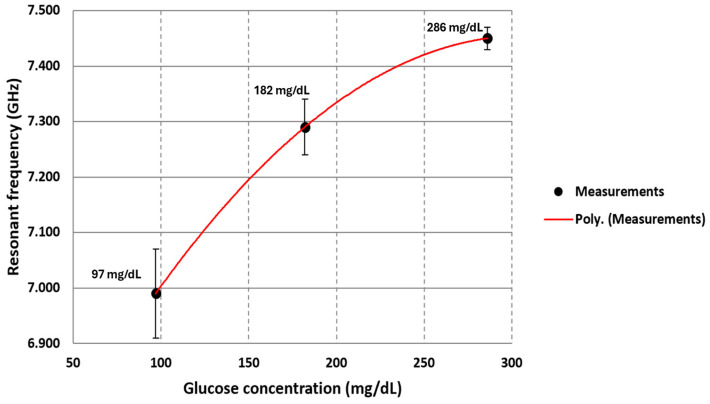
Correlation between the measured resonant frequency and glucose concentration. Error bars represent the mean ± standard deviation (SD) obtained from three independent repeated measurements.

Figure 5 presents the measured resonant frequency of the proposed SASR-D sensor as a function of glucose concentration, with error bars representing the mean ± standard deviation (SD) obtained from three repeated measurements. The results demonstrate a clear increase in resonant frequency from approximately 6.99 GHz at 97 mg/dL to 7.45 GHz at 286 mg/dL, confirming the sensor’s ability to distinguish different glucose concentrations. This behaviour is consistent with the reduction in the dielectric permittivity of the glucose solution as the glucose concentration increases, thereby shifting the resonant frequency toward higher values. The standard deviation of the resonant frequency was approximately 80, 50, and 20 MHz at 97, 182, and 286 mg/dL, respectively (*n* = 3). The largest measurement variation is observed at 97 mg/dL, which is attributed to the greater spreading and positional variability of the low-concentration droplet within the sensing region. As the glucose concentration increases, the droplet becomes slightly more stable due to the increased solution viscosity, resulting in improved measurement repeatability and reduced experimental variation. The metrological performance of the proposed sensor was further quantified in terms of repeatability, resolution, limit of detection, and measurement uncertainty. The standard deviation of the resonant frequency over three independent repeated measurements was approximately 80, 50, and 20 MHz at 97, 182, and 286 mg/dL, respectively, with the larger variation at low concentration attributed to the greater spreading and positional variability of the droplet within the sensing region.

The instrument-limited frequency resolution of the measurement, set by the VNA sweep of 201 points over 6.5–8 GHz, is 7.5 MHz, corresponding to a concentration resolution of approximately 3.1 mg/dL for the average sensitivity of 2.4 MHz/(mg/dL). The frequency resolution of the measurement is limited by the sampling of the vector network analyser, which acquired 201 points over the 6.5–8 GHz span. This corresponds to a minimum resolvable frequency shift of

*Δf_min_* = 1500 MHz/201 ≈ 7.5 MHz.

Dividing this minimum resolvable frequency shift by the average sensitivity of the sensor S = 2.4 MHz/(mg/dL), yields an instrument-limited concentration resolution of

*Δf_min_/S* = 25/2.4 ≈ 3.1 mg/dL.

Taking the measurement noise into account, the limit of detection, defined as 3σ divided by sensitivity, ranges from approximately 25 mg/dL at high concentration to 100 mg/dL at low concentration. The combined standard uncertainty of the retrieved concentration, obtained as the root-sum-of-squares of the repeatability and the VNA frequency-step contribution and propagated through the sensitivity, is ±33 mg/dL at 97 mg/dL and ±9 mg/dL at 286 mg/dL, dominated in all cases by droplet-handling repeatability rather than instrument noise.

Notably, the relationship between resonant frequency and glucose concentration is non-linear, exhibiting a steeper gradient at lower concentrations (97–182 mg/dL) and a progressively flattening response at higher concentrations (182–286 mg/dL). A non-linear saturation behaviour observed at higher glucose concentrations is consistent with the known response of metamaterial resonator sensors [28].

**Figure 6:**
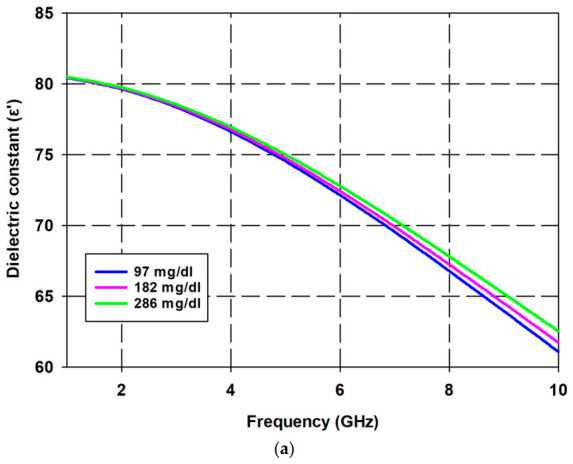

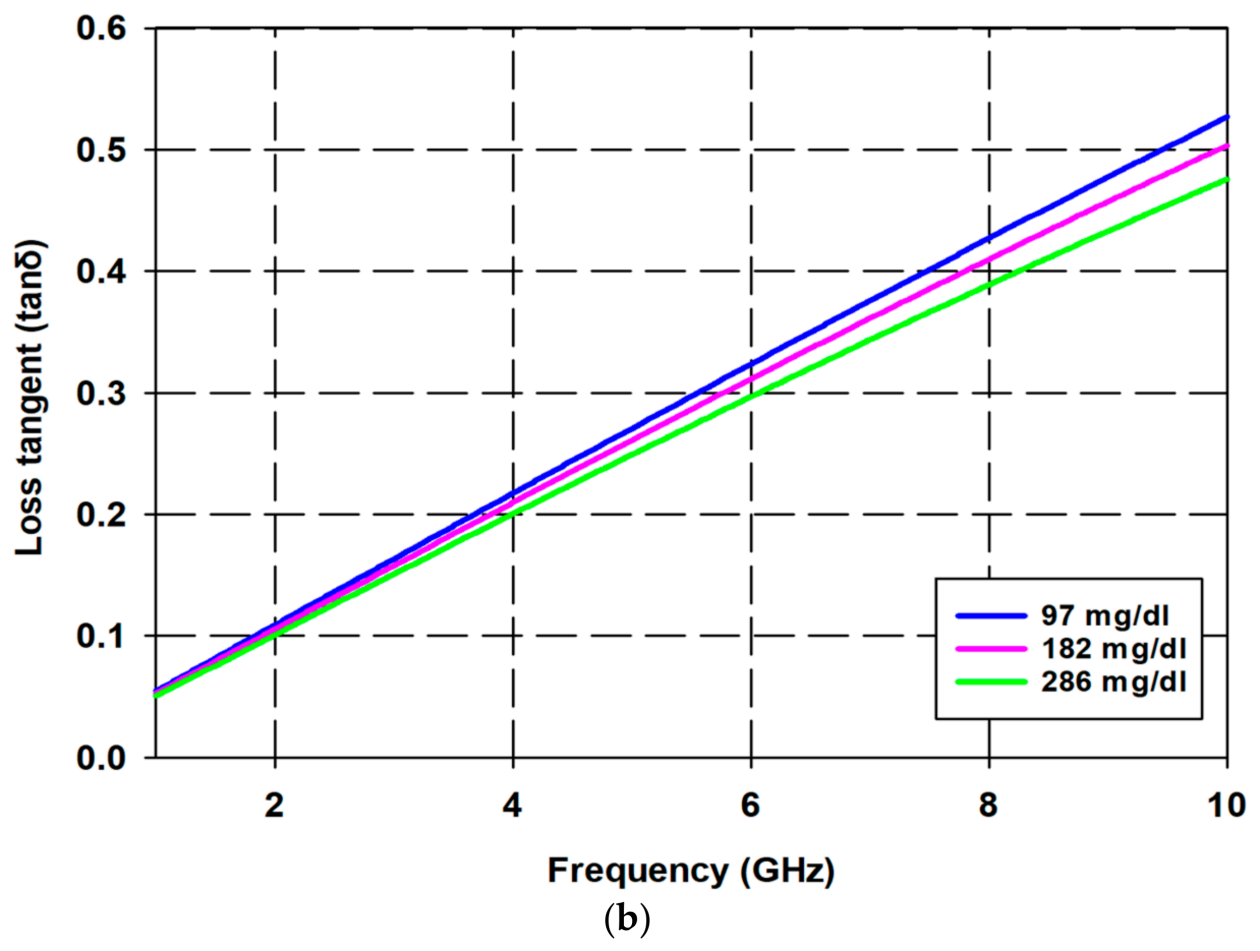
Calculated (**a**) dielectric constant and (**b**) loss tangent as a function of frequency.

The Debye parameters of the measured glucose solutions were extracted using Equation (1) and are summarised in Table 1. The dielectric constant and loss tangent as functions of frequency were then calculated using Equations (2)–(4) and are presented in Figure 6a and Figure 6b, respectively.

### 4.2. SASR-S Sensor

The single semi-circular resonator (SASR-S), shown in Figure 1a, was designed based on the architecture reported in [19], with the substrate material replaced by Rogers AD350A to achieve a higher Q-factor, as discussed previously.

**Figure 7:**
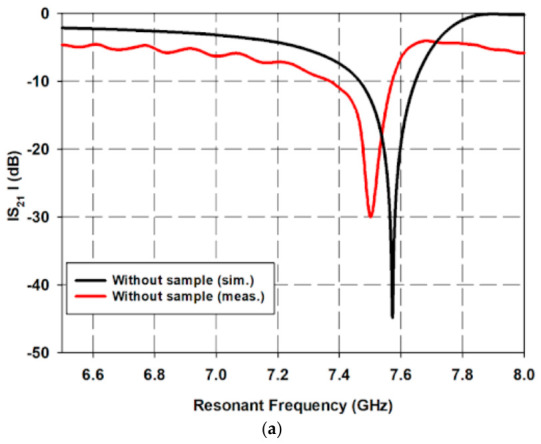

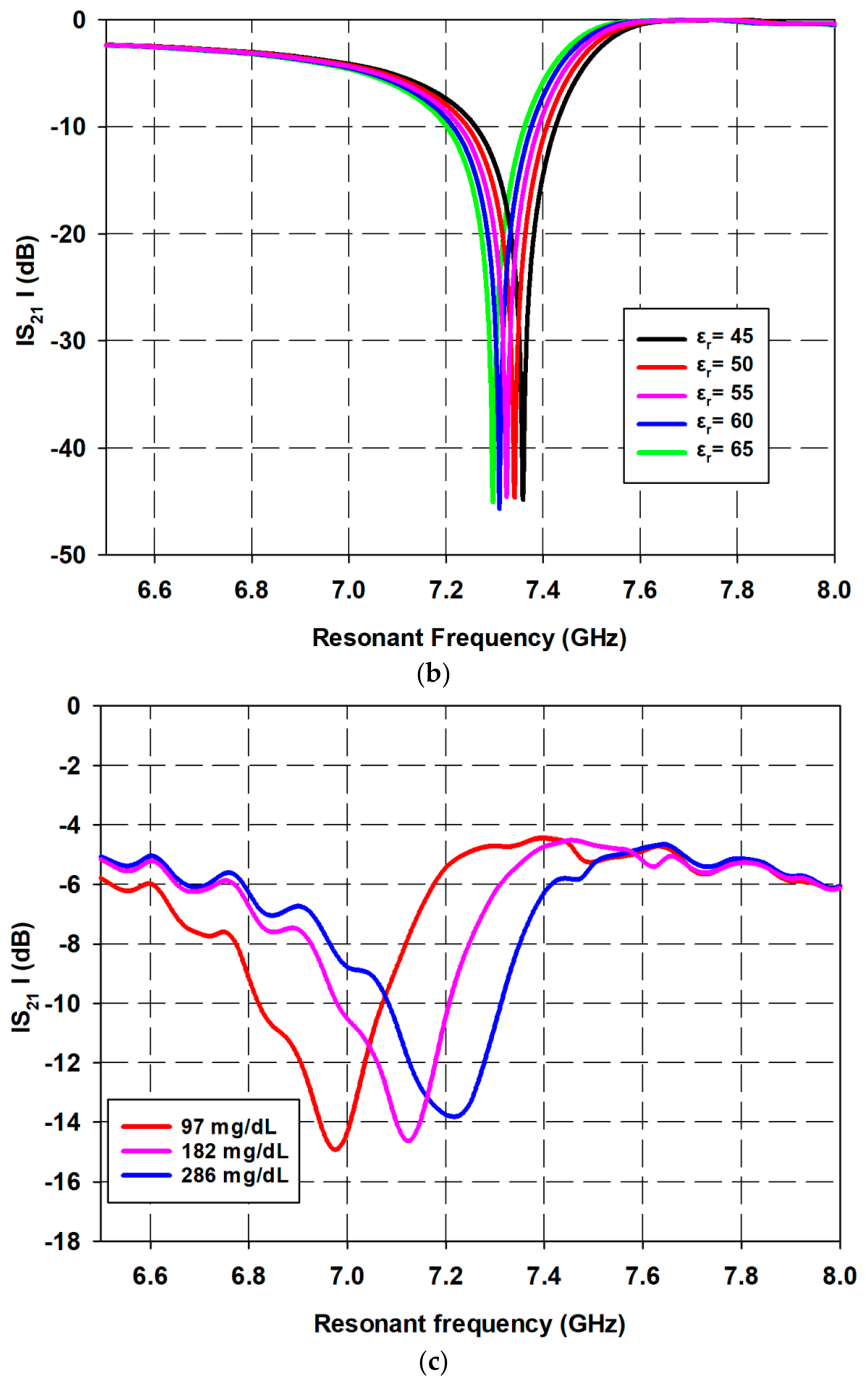
(**a**) The simulated and measured S_21_ of the sensor (unloaded). (**b**) The simulated S_21_ of the Sensor with different permittivity. (**c**) The measured S_21_ of the Sensor with different glucose concentrations.

Under unloaded-simulation conditions, as illustrated in Figure 7a, the resonator exhibited a resonant frequency of 7.57 GHz and an S_21_ magnitude of −44 dB. Corresponding experimental measurements conducted under unloaded conditions yielded a resonant frequency of 7.50 GHz and a shallower S21 response of −30 dB, representing a 0.92% deviation from the simulated value. The reduction in resonance depth between simulation and measurement is attributed to fabrication tolerances and connector losses.

Simulations were performed over the real permittivity range $\varepsilon^{\prime }$ = 45–65, as illustrated in Figure 7b. The results reveal a resonance frequency shift from 7.29 GHz to 7.36 GHz, corresponding to a total shift of 70 MHz.

Experimental measurements, shown in Figure 7c, were conducted across a glucose concentration range of 97 to 286 mg/dL. A total resonance frequency shift of 240 MHz (6.97 GHz to 7.21 GHz) was recorded, with the sensor achieving a sensitivity of 1.27 MHz/(mg/dL). These results confirm that the SASR-S configuration retains effective glucose sensing functionality. However, its performance is lower than that of the SASR-D sensor, as evidenced by a smaller frequency shift (240 MHz vs. 460 MHz) and reduced sensitivity. This performance improvement arises because the SASR-D configuration exploits both gap regions as active sensing areas, enabling a greater proportion of the confined electric field to interact simultaneously with the glucose sample, thereby producing a larger dielectric perturbation and a more pronounced resonant-frequency shift.

Sensitivity values are expressed in MHz/(mg/dL) using Equation (14) [9,19,31].

(14) $$S=\frac{\Delta f}{\Delta c}\mathrm{MHz}/(\mathrm{mg}/\mathrm{dL})$$

where:

S: Sensitivity;

∆c: Concentration changes in glucose;

∆f: Frequency shift.

Although both simulation and experimental results demonstrate the superior performance of the SASR-D design, some discrepancies were observed between the simulated and measured results. These differences arise primarily from the idealised conditions assumed in the numerical model. In the CST simulations, the glucose sample was represented by fixed dimensions that precisely matched the semicircular sensing region, and its position was kept constant across all glucose concentrations. In contrast, the experimental measurements were subject to unavoidable practical variations. The glucose solutions were prepared manually, and all measurements were performed immediately after mixing to minimise any potential concentration variations over time. The glucose droplet was manually positioned within the sensing region, resulting in slight variations in its exact location. Furthermore, small differences in spreading behaviour were observed between measurements, particularly for the 97, 182, and 286 mg/dL glucose solutions. These variations can affect the effective electromagnetic interaction between the sample and the resonator, leading to changes in the resonant-frequency shift and, consequently, deviations between the simulated and measured results.

Despite these uncertainties, the measured results provide proof-of-concept validation of the proposed dual-hotspot approach’s effectiveness. The SASR-D sensor exhibited approximately twice the sensitivity of the single semi-circular SASR-S configuration. Importantly, this enhancement was achieved without introducing additional resonator elements or increasing the overall sensor footprint. These findings demonstrate that introducing a second sensing hotspot significantly enhances the interaction between the electromagnetic field and the glucose sample, resulting in a larger resonance-frequency shift and improved sensitivity. Table 2 compares the performance of the proposed sensor with previously reported microwave glucose sensors.

## 5. Conclusions

This paper presented a C-band microwave biosensor based on an asymmetric split-ring resonator in two configurations: a single semi-circular (SASR-S) design and a proposed double semi-circular (SASR-D) design. The introduction of a second semi-circular sensing element in the SASR-D configuration increased the effective sensing area, resulting in a measured resonance-frequency shift of 460 MHz and a sensitivity of 2.4 MHz/(mg/dL) over a glucose concentration range of 97–286 mg/dL at a controlled room temperature of 25 °C. This represents an approximately 1.9-fold improvement over the SASR-S configuration, which exhibited a resonance-frequency shift of 240 MHz and a sensitivity of 1.27 MHz/(mg/dL) under the same experimental conditions.

The high Q-factor of 408, achieved using the Rogers AD350A substrate, contributed to the sharp response and improved frequency-shift detection. Comparison with previously reported microwave glucose sensors demonstrates that the proposed SASR-D sensor offers competitive sensitivity while maintaining a compact footprint and requiring only a 2 µL volume of sample.

The experimental results provide proof-of-concept validation of the proposed sensing approach. The measurements confirm the sensitivity enhancement achieved by simultaneously utilising the two inherent sensing hotspots of the asymmetric split-ring resonator, in agreement with the electromagnetic simulation results. However, a comprehensive experimental characterization over a wider range of glucose concentrations, together with selectivity studies in complex biological media and further metrological evaluation, will be the subject of future work.

## Figures and Tables

**Table 1:** Extracted Debye coefficients for the investigated glucose concentrations.

Glucose (mg/dL)	$\varepsilon_{\infty }$	$\varepsilon_{stat}$	τ (ps)
97	8.29	80.7001	9.70231
182	10.84	80.7177	9.72186
286	13.96	80.7392	9.74578

**Table 2:** Comparison of the proposed sensor with previously reported glucose sensors.

Ref.	Sensing Technique	Frequency (GHz)	Sensitivity (MHz/(mg/dL))	Type of Substrate	Q-Factor	Normalized Sensitivity (%/(mg/dL)) ^a^	Sensing Parameter	Concentration (mg/dL)	Sample Volume (μL)
[32]	Microstrip resonator	6.5	3.13 × 10^−2^	FR-4	27	4.8 × 10^−4^	S_21_	20,000–30,000	50
[33]	Ground-signal-ground LC	1–4.5	0.26	silicon oxide	NR	9.5 × 10^−3^	S_21_	0–72	NR
[34]	Dual-band microwave transmission-line sensor	2.4–2.5	2	FR-4	16.3	8.2 × 10^−2^	S_21_	80–170	NR
[35]	Concentric SRR-based microwave biosensor	2.76–3.25	1.964	FR-4	20	6.5 × 10^−2^	S_11_	0–400	NR
[15]	DS-CSRR	3.3	1.9 × 10^−3^	FR-4	130	5.8 × 10^−5^	S_21_	0–18,000	600
This work	Double Semi-circular resonator developed in this paper	7.56	2.4	Rogers AD350A	408	3.2 × 10^−2^	S_21_	97–286	2

NR: not reported in the cited reference. ^a^ Normalized sensitivity defined as (Δf/ΔC)/f_0_ × 100.

## Data Availability

The original contributions presented in this study are included in the article. Further inquiries can be directed to the corresponding author.
